# Maternal immune activation in mice recapitulates features of attention-deficit/hyperactivity disorder (ADHD) in susceptible offspring

**DOI:** 10.1038/s41386-026-02373-7

**Published:** 2026-02-28

**Authors:** Ron Schaer, Nicole Wenger, Sarah Steiner, Tina Notter, Urs Meyer

**Affiliations:** 1 Institute of Veterinary Pharmacology and Toxicology, University of Zurich, Zurich, Switzerland; 2 Institute of Pharmacology and Toxicology, University of Zurich, Zurich, Switzerland; 3 Neuroscience Center Zurich, University of Zurich and ETH Zurich, Zurich, Switzerland

**Keywords:** Developmental disorders, Neuroimmunology

## Abstract

Prenatal exposure to infectious or non-infectious maternal immune activation (MIA) represents a transdiagnostic environmental risk factor for psychiatric and neurodevelopmental disorders. Building on previous findings of locomotor hyperactivity in a subset of male MIA offspring, the present study investigated whether viral-like MIA in mice recapitulates features of attention-deficit/hyperactivity disorder (ADHD) in this subgroup. We show that 40–50% of MIA-exposed male offspring develop locomotor hyperactivity in a novel environment, which is most pronounced during early- to mid-adolescence and precedes the emergence of increased impulsive behavior and pre-attentive filtering deficits in early adulthood. We further identified subgroup-specific dopaminergic and noradrenergic alterations in cortical and subcortical brain regions of MIA offspring. These neuronal alterations were age-dependent and correlated with behavioral changes. Moreover, treatment with methylphenidate (MPH), a first-line pharmacological therapy for ADHD, normalized locomotor hyperactivity and restored abnormal mesolimbic and striatal activation patterns in susceptible MIA offspring. Collectively, our findings demonstrate that MIA in mice recapitulates key features of ADHD in a susceptible subset of offspring, supporting the notion that MIA may contribute etiologically to ADHD in some individuals. More broadly, our results suggest that the heterogeneous neurobehavioral outcomes of MIA offspring may result from distinct yet overlapping pathophysiological mechanisms across neurodevelopmental and psychiatric disorders.

## Introduction

Prenatal exposure to infectious or non-infectious maternal immune activation (MIA) represents a transdiagnostic environmental risk factor for psychiatric and neurodevelopmental disorders [1–4]. Despite converging evidence for the significant health impacts of MIA, its outcomes in the offspring remain heterogeneous across both sexes [5–8]. Thus, MIA does not uniformly produce central nervous system abnormalities in all offspring, suggesting the presence of substantial resilience or plasticity to its effects. Comparable phenotypic heterogeneity has also been observed in preclinical models, where MIA induces variable outcomes even under rigorously standardized experimental conditions [9–13].

Using longitudinal experimental designs in a mouse model of MIA, we recently demonstrated that offspring can be stratified into distinct subgroups based on their early behavioral trajectories [14]. During early adolescence, MIA-exposed offspring segregated into phenotypically divergent clusters characterized by distinct behavioral profiles in a sex-dependent manner [14]. A notable finding was that a subset of susceptible MIA offspring (SUS-MIA) exhibited pronounced locomotor hyperactivity during early adolescence, whereas resilient counterparts (RES-MIA) showed no such alterations. This hyperactive phenotype emerged specifically in male SUS-MIA offspring and predicted the later development of additional behavioral abnormalities in adulthood, including deficits in sensorimotor gating and impairments in social interaction [14].

The emergence of a hyperactive phenotype in a subset of SUS-MIA offspring prompted us to examine whether MIA in mice may recapitulate features of attention-deficit/hyperactivity disorder (ADHD). ADHD is a neurodevelopmental disorder affecting 3–8% school-aged children globally, with symptoms persisting into adulthood in more than 60% of cases [15–18]. It is characterized by varying degrees of pervasive inattention, hyperactivity, and impulsivity, and is associated with substantial functional impairment in social, occupational, and academic domains [15–18]. Notably, epidemiological evidence shows that ADHD frequently co-occurs with other psychiatric and neurodevelopmental disorders, including schizophrenia, depression, and autism spectrum disorder (ASD) [19–23]. Moreover, childhood ADHD has been identified as a risk factor for developing schizophrenia later in life [22–24]. These findings suggest that nosologically defined neurodevelopmental and psychiatric disorders may lie along a continuum of neurodevelopmental causalities, in which the combination of genetic architecture and environmental context shapes the specificity of pathological outcomes [25–29]. Within this framework, MIA may act as a risk factor with transdiagnostic etiological relevance. However, the epidemiological link between MIA and ADHD remains equivocal, with some studies reporting a significant association [30–36] and others failing to detect a consistent relationship [37–40]. This inconsistency raises the possibility that MIA produces ADHD-relevant dysfunctions in only a subgroup of offspring, highlighting the need for preclinical investigations to define mechanisms underlying this selective effect [41].

Therefore, this study examined whether MIA in mice reproduces behavioral and neuronal phenotypes of ADHD in susceptible offspring displaying hyperactivity during early adolescence (SUS-MIA offspring), using control (CON) and resilient MIA offspring lacking hyperactivity (RES-MIA offspring) as comparators. First, we conducted longitudinal analyses to characterize the trajectory of subgroup-specific locomotor hyperactivity from the early adolescent to adult stage and its association with pre-attentive filtering in early adulthood. The latter was assessed using a paradigm of prepulse inhibition (PPI) of the acoustic startle reflex, an operational measure of pre-attentative filtering known to be affected by MIA [1, 9, 14]. Second, we assessed whether subgroup-specific hyperactivity was associated with deficits in reward processing and impulsivity, two behavioral domains strongly linked to ADHD [42–49]. Third, we compared neuronal alterations in subgroups of MIA and CON offspring, with a particular focus on dopaminergic and noradrenergic systems implicated in ADHD [50–55]. Finally, we examined whether treatment with methylphenidate (MPH), a first-line pharmacological treatment for ADHD [56, 57], could normalize subgroup-specific locomotor hyperactivity. Akin to our previous investigations [9, 12, 14], all experiments were conducted using a mouse model of viral-like MIA, induced via prenatal administration of the synthetic double-stranded RNA analog poly(I:C) (*polyriboinosinic–polyribocytidylic acid*). Based on prior findings showing that adolescent hyperactivity emerges in a subset of male but not female offspring [14], all experiments were performed exclusively in male MIA offspring and corresponding controls.

## Materials and methods

### Animals

C57BL/6N male and female breeder mice (12 weeks old) were obtained from Charles River Laboratories (Sulzfeld, Germany) and housed in individually ventilated cages (IVCs; Allentown Inc., Bussy-Saint-Georges, France) as previously described [58]. Mice were maintained in a specific-pathogen-free facility with controlled temperature (21 ± 3 °C) and humidity (50 ± 10%) under a reversed 12 h light–dark cycle (lights off 9:00 AM–9:00 PM), with *ad libitum* access to standard chow (Kliba 3336, Kaiseraugst, Switzerland) and water. All procedures were approved by the Cantonal Veterinarian's Office of Zurich, Switzerland.

### Breeding and maternal manipulations

C57BL/6N mice were used throughout the study. Animal housing and maintenance are described in the *Supplementary Information*. Timed pregnancies were established via in-house breeding, initiated 2 weeks after breeder animals were acclimated to the facility (*Supplementary Information*). On gestation day (GD) 12, pregnant mice were randomly assigned to a single intraperitoneal (i.p.) injection of low molecular weight poly(I:C) (10 mg/kg; InvivoGen, Toulouse, France; cat.#: tlrl-picw, lot #PIW-41–05) or pyrogen-free 0.9% NaCl vehicle (B. Braun, Melsungen, Switzerland), using an injection volume of 10 ml/kg. The quality, molecular composition, and immunopotency of this poly(I:C) lot were previously validated [59, 60]. Immediately after injection, dams were returned to their home cages and left undisturbed until 5 days postpartum. Four cohorts of dams were generated via identical on-site breeding procedures and maternal treatments (Supplementary Table 1). Overall, approximately twice as many MIA dams as CON dams were prepared, as we anticipated that ~50% of the offspring would be allocated to the RES-MIA and SUS-MIA subgroups, respectively, based on stratification using behavioral data from adolescent offspring [14]. Further methodological details on maternal manipulations are provided in the *Supplementary MIA Reporting Guidelines* [61].

### Locomotor activity tests

A standard open field test was used to assess locomotor activity [62]. Detailed descriptions of the apparatus and testing procedures are provided in the *Supplementary Information*. To evaluate baseline locomotor activity and stratify MIA offspring into SUS-MIA and RES-MIA subgroups based on locomotor activity during early adolescence [14], each CON and MIA offspring was allowed to freely explore the open field for 10 min on postnatal day (PND) 28. For longitudinal assessment of locomotor activity, the open field test was performed weekly starting on PND 28 until early adulthood, with the final test conducted on PND 63. To assess the acute effects of MPH on locomotor activity, the animals were pretreated with a single dose of MPH (LGC Standards, Wesel, Germany; cat. #TRC-M325880) or vehicle (VEH) 60 min before locomotor activity testing, and the open field session lasted 30 min. MPH was dissolved in a 30% condensed milk solution, maintained under continuous agitation throughout the procedure, and administered orally via the micropipette-guided drug administration method, as described previously [63–65]. MPH was administered at a dose of 2 mg/kg in a volume of 2 ml/kg. This dose was selected based on prior studies demonstrating that MPH at dose ranges of 1–3 mg/kg normalizes locomotor hyperactivity in ADHD-relevant mouse models without significantly affecting locomotor behavior in control mice [66–68]. Animals assigned to VEH treatment received an equivalent volume of the 30% condensed milk solution.

### Reward processing and impulsivity tests

Reward processing and impulsivity were assessed using the IntelliCage system (TSE Systems, Bad Homburg, Germany), a fully automated home-cage apparatus designed to evaluate a wide range of behaviors in group-housed mice tagged with radio-frequency identification microtransponders [69–72]. Methodological details of the IntelliCage system are provided in the *Supplementary Information*.

After completion of baseline locomotor activity testing in the open field (see above), mice assigned to the assessment of reward processing and impulsivity in the IntelliCage system received a subcutaneous microtransponder under isoflurane anesthesia once they reached PND 56, as detailed in the *Supplementary Information*. Following transponder implantation, mice were transferred from conventional IVCs (see above) to the IntelliCages in groups of 8–10 animals per cage and habituated for 7 days (PNDs 56–63; *Supplementary Information*). After the habituation phase, a nosepoke adaptation phase was conducted over three consecutive days, as described in the *Supplementary Information*. Upon completion of habituation and nosepoke adaptation, testing for reward processing and impulsivity commenced and included the following tasks: (i) sucrose preference test to assess hedonic responses to a natural reward [73], (ii) progressive ratio test to measure incentive motivation to obtain a natural reward (sucrose solution) [74–76], and (iii) operant delay-of-reinforcement task to evaluate impulsivity and delay tolerance in obtaining a natural reward (sucrose solution) [77–79]. Detailed methodological descriptions for each test are provided in the *Supplementary Information*.

### Pre-attentive filtering test

Pre-attentive filtering was assessed using a test of PPI of the acoustic startle reflex, in which the reflexive startle response to an intense acoustic pulse stimulus is attenuated when its presentation is shortly preceded by a weak prepulse stimulus [80]. Methodological details of the PPI test are provided in the *Supplementary Information*.

### Immunohistochemistry and microscopy

Animals assigned to the immunohistochemical investigations were transcardially perfused under deep anesthesia (*Supplementary Information*). Immunofluorescent stainings were performed according to previously established protocols [14, 64, 81, 82], as described in the *Supplementary Information*. For the evaluation of dopaminergic and noradrenergic markers, the following primary antibodies were used: rabbit anti-tyrosine hydroxylase (TH; Merck Millipore, cat. # AB152, 1:2000), rabbit anti-dopamine-β-hydroxylase (DBH; Abcam, cat. # ab209487, 1:1000), rat anti-dopamine transporter (DAT; Merck Millipore, cat. # MAB369, 1:1000), and mouse anti-norepinephrine transporter (NET; MAb Technologies Inc., cat. # NET05-2, 1:5000). DAT and NET were selected based on previous findings showing altered expression levels of these transporters in the mesocorticolimbic and striatal regions of individuals with ADHD [51, 54, 83], as well as in ADHD-relevant animal models [84–87]. TH and DBH were included because they are the critical enzymes for dopamine and norepinephrine synthesis in vivo [88, 89] and have been reported to be affected by MIA [90–92], with recent studies indicating subgroup- and sex-specific effects [14]. In addition, immunohistochemical analyses of c-Fos expression were conducted to assess neuronal activation patterns [64, 81] 90 min after MPH or VEH treatment. For this purpose, a rabbit anti-c-Fos primary antibody (Santa Cruz Biotechnology, cat. # sc-52, 1:1000) was used. All immunofluorescent preparations were imaged and quantified in the medial prefrontal cortex (mPFC, Bregma: +2.0 to + 1.6 mm), nucleus accumbens (NAc; Bregma: +1.7 to + 0.9 mm), and caudate putamen (CPu; Bregma: +1.2 to + 0.4 mm) using an automated upright widefield slide scanning microscope (PhenoImager™ HT, Akoya Biosciences, Marlborough, USA), as described in the *Supplementary Information*. However, given the sparse expression of DBH and NET in the CPu and NAc [89, 93], these markers were only quantified in the mPFC, where they are abundantly expressed [94, 95].

### Statistical analysis

All statistical analyses were performed using SPSS Statistics (version 30.0; IBM, Armonk, NY, USA) and Prism (version 10.0; GraphPad Software, La Jolla, CA, USA), with statistical significance set at *p* < 0.05 unless specified otherwise. Unsupervised two-step cluster analysis was used to stratify MIA offspring into SUS-MIA and RES-MIA subgroups based on differences in baseline locomotor activity during early adolescence (PND 28), as previously described [9, 11, 14]. Following the stratification of CON and MIA offspring, one-way analysis of variance (ANOVA) or repeated-measures ANOVA (RM-ANOVA) was conducted to analyze dependent variables across tests, followed by Tukey’s post hoc test for multiple comparisons where appropriate. In addition, one-sample *t*-tests with a hypothetical value of 50% were used to ascertain whether the sucrose preference scores were significantly above the chance level of 50%. Relationships between locomotor activity and immunohistochemical markers were analyzed using Pearson’s product-moment correlations, with Bonferroni correction applied for multiple comparisons.

## Results

### Developmental trajectory of locomotor hyperactivity in susceptible MIA offspring and its association with pre-attentive filtering in early adulthood

**Fig. 1:**
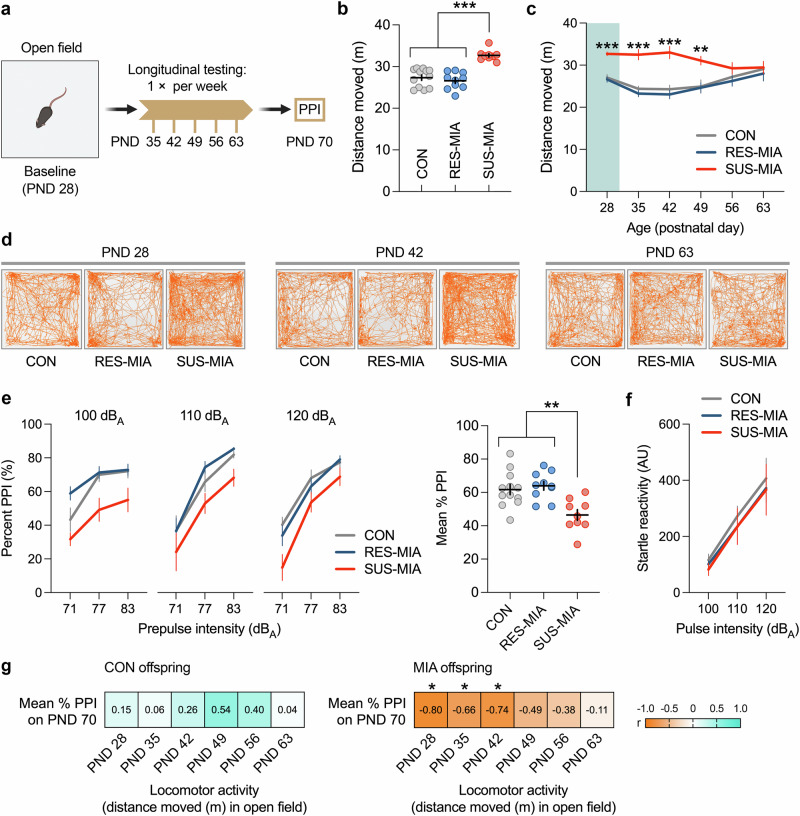
Developmental trajectory of locomotor activity and its association with adult pre-attentive filtering in control (CON) offspring and subgroups of offspring exposed to maternal immune activation (MIA). **a** Schematic representation of the behavioral testing sequence. On postnatal day (PND) 28, offspring underwent an initial open field test to stratify MIA offspring into resilient (RES-MIA) and susceptible (SUS-MIA) subgroups based on locomotor activity scores during early adolescence. The same CON and MIA subgroups were subsequently re-tested in the open field once per week, with the final open field test conducted on PND 63. On PND 70, the animals were subjected to a prepulse inhibition (PPI) of the acoustic startle response test to assess pre-attentive filtering. **b** Distance moved in the baseline open field test on PND 28 for CON and MIA subgroups, confirming locomotor hyperactivity in the SUS-MIA subgroup during early adolescence. **c** Distance moved in repeated open field tests conducted weekly from early adolescence to adulthood (PNDs 28–63). The turquoise shading indicates baseline locomotor activity scores obtained from the open field test on PND 28. **d** Representative computer-generated path drawings illustrating the locomotor trajectories of CON and MIA subgroups in the open field at successive developmental stages (PND 28, 42, and 63). **e** Line plots show percent PPI (%) as a function of prepulse intensity (71, 77, and 83 dB_A_) for each of the three pulse conditions (100, 110, and 120 dB_A_). The scatter plot displays the mean % PPI across all prepulse and pulse intensities. **f** The line plot depicts acoustic startle reactivity (in arbitrary units, AU) across the three pulse conditions (100, 110, and 120 dB_A_). **g** Pearson’s product-moment correlations between mean % PPI on PND 70 and locomotor activity scores (total distance moved in the open field) assessed for CON and MIA offspring across successive developmental stages (PND 28-63). Negative and positive correlations are represented in orange and mint green color, respectively. Significant correlations are denoted with the symbol (*) after correction for multiple comparisons (significance threshold: ^*^p ≤ 0.0084). All line plots represent means ± s.e.m., and scatter plots show individual data points with overlaid group means ± s.e.m.; ^**^p < 0.01, ^***^p < 0.001, based on Tukey’s post hoc test following ANOVA or RM-ANOVA.

Using the first cohort of offspring (Supplementary Table 1), we examined the trajectory of subgroup-specific locomotor hyperactivity from early adolescence to adulthood. Offspring were initially stratified into subgroups based on their performance in the open field test on PND 28 (Fig. 1a). Locomotor activity within these same subgroups was then assessed weekly, with the final open field test conducted on PND 63 (Fig. 1a). Two-step cluster analysis of the total distance moved in the baseline open field on PND 28 test demonstrated that 47% (9 out of 19) of MIA offspring exhibited locomotor hyperactivity compared to CON offspring, whereas the remaining 53% (10 out of 19) did not (ANOVA: *F*_(2,28)_ = 28.26, *p* < 0.001; SUS-MIA vs. RES-MIA or CON: *p* < 0.001; Fig. 1b). SUS-MIA offspring exhibiting overall locomotor hyperactivity also showed subgroup-specific increases in the distance moved in the center zone of the open field and the time spent in the center zone (Supplementary Fig. S1).

Longitudinal assessment of locomotor activity revealed distinct developmental trajectories among SUS-MIA, RES-MIA, and CON offspring (Fig. 1c, d). While CON and RES-MIA offspring exhibited a U-shape-like pattern of locomotor activity across development, SUS-MIA offspring showed persistently elevated activity between postnatal days (PND) 28 and 49, followed by a decline, such that by PND 56, locomotor activity levels were comparable across subgroups (Fig. 1c, d). These findings were supported by RM-ANOVA of locomotor activity, revealing a significant main effect of age (*F*_(5,140)_ = 2.39, *p* < 0.05) and its interaction with subgroup (*F*_(10,140)_ = 2.83, *p* < 0.01). Post hoc comparisons confirmed the significant differences between SUS-MIA and RES-MIA or CON offspring on PNDs 28 (*p* < 0.001), 35 (*p* < 0.001), 42 (*p* < 0.001), and 49 (*p* < 0.01).

Upon completion of the longitudinal assessment of locomotor activity, the same subgroups were subjected to a PPI test of the acoustic startle reflex to measure pre-attentative filtering on PND 70 (Fig. 1a). Compared to CON and RES-MIA offspring, SUS-MIA offspring exhibited a significant reduction in PPI scores (Fig. 1e). This reduction was notable across all pulse intensities (100, 110, and 120 dB_A_), leading to a significant main effect of subgroups in RM-ANOVA of % PPI (*F*_(2,28)_ = 9.19, *p* < 0.001). Post hoc analysis of the mean % PPI confirmed the significant differences between SUS-MIA and RES-MIA or CON offspring (*p*’s < 0.01). Acoustic startle responses to pulse-alone trials did not differ among subgroups (Fig. 1f), indicating that the subgroup-specific reductions in %PPI reflected genuine impairments in pre-attentive filtering rather than alterations in baseline startle reactivity.

Possible associations between locomotor activity and PPI were examined using Pearson’s product-moment correlations, which were performed separately for CON and MIA offspring and corrected for multiple comparisons (significance threshold: *p* ≤ 0.0084). Significant negative correlations were observed in MIA offspring between mean % PPI and locomotor activity on PND 28 *(r* = −0.80, *p* < 0.001), 35 (*r* = −0.66, *p* < 0.003) or 42 (*r* = −0.74, *p* < 0.001), whereas no such correlations were found in CON offspring (Fig. 1g). Thus, higher locomotor activity during early- to mid-adolescence was associated with poorer pre-attentive filtering in adulthood following prenatal MIA exposure.

### Susceptible MIA offspring show increased impulsivity in an operant delay-of-reinforcement task

**Fig. 2:**
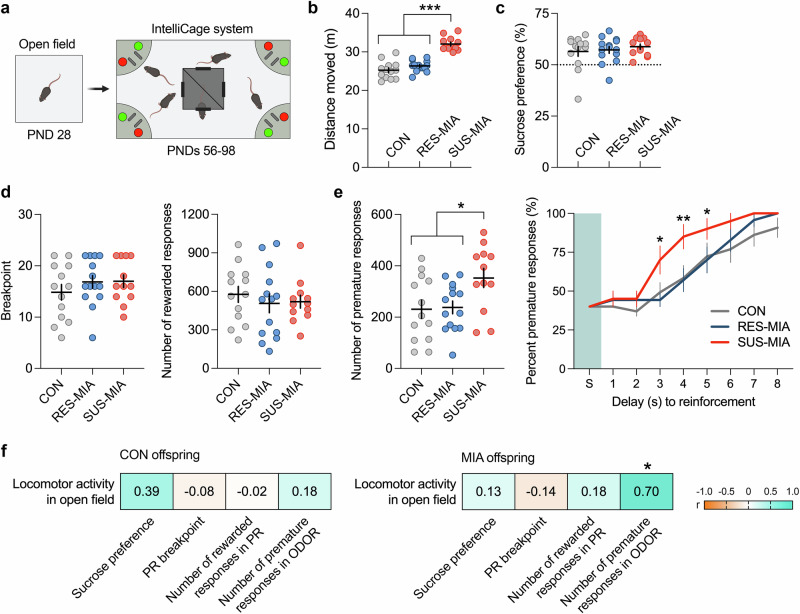
Reward processing and impulsivity in control (CON) offspring and subgroups of offspring exposed to maternal immune activation (MIA). **a** Schematic representation of the behavioral testing sequence. On postnatal day (PND) 28, offspring were subjected to an open field test to stratify MIA offspring into resilient (RES-MIA) and susceptible (SUS-MIA) subgroups based on locomotor activity scores during early adolescence. The same CON and MIA subgroups were subsequently tested in the IntelliCage system to assess reward processing and impulsivity between PNDs 56 and 98. **b** Distance moved in the open field test for CON and MIA subgroups, confirming the presence of locomotor hyperactivity in the SUS-MIA subgroup during early adolescence. **c** Percent sucrose consumption in the sucrose preference test for CON and MIA subgroups conducted in the IntelliCage system. The dashed line represents the chance level (50%). **d** Breakpoint and number of rewarded responses for CON and MIA subgroups in the progressive ratio test conducted in the IntelliCage system. **e** Total number of premature responses and percent premature responses as a function of increasing delays to reinforcement in the operant delay-of-reinforcement test conducted in the IntelliCage system. The turquoise shading denotes the shaping (S) phase conducted with a minimal delay of 0.5 s. **f** Pearson’s product-moment correlations between baseline locomotor activity (total distance moved in the open field) and behavioral measures of reward processing and impulsivity for CON and MIA offspring. Negative and positive correlations are represented in orange and mint green color, respectively. Significant correlations are denoted with the symbol (*) after correction for multiple comparisons (significance threshold: ^*^p ≤ 0.0125). All scatter plots show data points from individual mice with overlaid group means ± s.e.m.; ^*^p < 0.05, ^**^p < 0.01, ^***^p < 0.001, based on Tukey’s post hoc test following ANOVA or RM-ANOVA. ODOR, operant delay-of-reinforcement; PR, progressive ratio.

Using the second cohort of offspring (Supplementary Table 1), we investigated whether MIA leads to ADHD-related deficits in reward processing and impulsivity in a subgroup-specific manner (Fig. 2a). In this cohort, the initial assessment of baseline locomotor activity in the open field on PND 28 showed that 46% (12 out of 26) of MIA offspring exhibited hyperactivity compared to CON offspring, whereas the remaining 54% (14 out of 26) did not (ANOVA: *F*_(2,36)_ = 49.15, *p* < 0.001; SUS-MIA vs. RES-MIA or CON: *p* < 0.001; Fig. 2b).

The subsequent assessment of reward processing in the IntelliCage system revealed no differences between CON and MIA offspring in sucrose preference, regardless of whether the MIA offspring belonged to the SUS-MIA or RES-MIA subgroups (Fig. 2c). One-sample *t*-tests with a hypothetical mean of 50% confirmed that sucrose preference scores were significantly above the chance level of 50% in all subgroups (CON: *t*_(12)_ = 2.77, *p* < 0.05; RES-MIA: *t*_(13)_ = 4.39, *p* < 0.001; SUS-MIA: *t*_(11)_ = 7.02, *p* < 0.001). Likewise, there were no differences between CON, RES-MIA, and SUS-MIA offspring with regard to behavioral measures assessed using a progressive ratio task. As shown in Fig. 2d, the breakpoint (i.e., the highest ratio of responses completed to obtain a reward before the animal ceased responding) as well as the total number of rewarded responses were identical across subgroups. Together, these findings demonstrate that MIA does not alter the hedonic responses or incentive motivation toward a natural reward.

However, MIA induced subgroup-specific alterations in behavioral indices of impulsivity for obtaining a natural reward, as assessed using an operant delay-of-reinforcement task. Specifically, while no differences were observed between CON and RES-MIA offspring, SUS-MIA offspring exhibited a significant increase in the total number of premature nosepoke responses, defined as any nosepoke occurring during the delay period between trial initiation and access to the reward (ANOVA: *F*_(2,36)_ = 4.27, *p* < 0.05; SUS-MIA vs. RES-MIA or CON: *p* < 0.05; Fig. 2e). The percentage of premature responses generally increased with longer delays (Fig. 2e), as indicated by the significant main effect of delay to reinforcement (*F*_(8,288)_ = 65.01, *p* < 0.001). Notably, compared to CON and RES-MIA offspring, SUS-MIA offspring displayed a significant elevation in premature responding at 3-, 4-, and 5-sec delay intervals. This effect was supported by a significant subgroup × delay interaction (*F*_(16,288)_ = 2.01, *p* < 0.05), with post hoc comparisons confirming higher percentages of premature responses in SUS-MIA offspring relative to CON or RES-MIA at the 3-sec (*p* < 0.05), 4-sec (*p* < 0.01), and 5-sec (*p* < 0.05) delays (Fig. 2e).

Pearson’s correlations, conducted separately for CON and MIA offspring and corrected for multiple comparisons (significance threshold: *p* ≤ 0.0125), revealed that the total number of premature nosepoke responses in the operant delay-of-reinforcement task correlated positively with baseline locomotor activity in MIA offspring (*r* = 0.70, *p* < 0.001), but not in CON offspring (Fig. 2f). No other correlations between locomotor activity and reward-related behaviors reached significance. These results indicate that higher locomotor activity was specifically associated with behavioral indices of impulsivity.

### Susceptible MIA offspring show distinct dopaminergic and noradrenergic alterations in cortical and subcortical brain regions during early adolescence

**Fig. 3:**
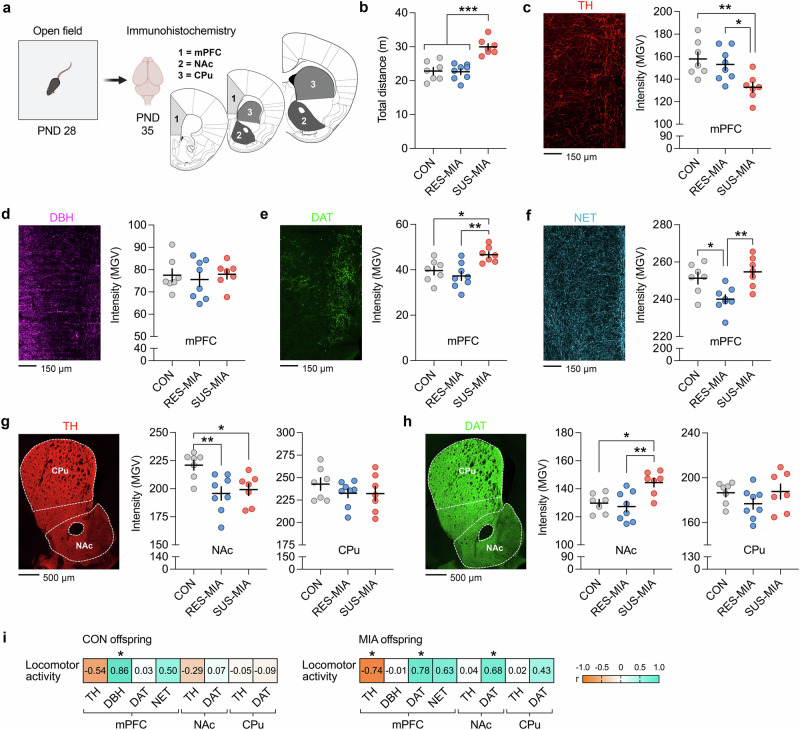
Early adolescent dopaminergic and noradrenergic markers in control (CON) offspring and subgroups of offspring exposed to maternal immune activation (MIA). **a** Schematic representation of the behavioral and immunohistochemical testing sequence. On postnatal day (PND) 28, offspring were subjected to an open field test to stratify MIA offspring into resilient (RES-MIA) and susceptible (SUS-MIA) subgroups based on locomotor activity scores during early adolescence. The same CON and MIA subgroups were then subjected to immunohistochemical analyses of dopaminergic and noradrenergic markers in the medial prefrontal cortex (mPFC), nucleus accumbens (NAc), and caudate putamen (CPu) when they reached PND 35. **b** Distance moved in the open field test for CON and MIA subgroups, confirming the presence of locomotor hyperactivity in the SUS-MIA subgroup during early adolescence. **c** Representative immunofluorescence stain of tyrosine hydroxylase (TH) and TH intensity (mean gray value, MGV) in the mPFC. **d** Representative immunofluorescence stain of dopamine-β-hydroxylase (DBH) and DBH intensity (MGV) in the mPFC. **e** Representative immunofluorescence stain of dopamine transporter (DAT) and DAT intensity (MGV) in the mPFC. **f** Representative immunofluorescence stain of norepinephrine transporter (NET) and NET intensity (MGV) in the mPFC. **g** Representative immunofluorescence stain of TH and TH intensities (MGV) in the NAc and CPu. **h** Representative immunofluorescence stain of DAT and DAT intensities (MGV) in the NAc and CPu. **i** Pearson’s product-moment correlations between locomotor activity scores (as assessed using the open field test on PND 28) and immunohistochemical markers for CON and MIA offspring. Negative and positive correlations are represented in orange and mint green color, respectively. Significant correlations are denoted with the symbol (*) after correction for multiple comparisons (significance threshold: ^*^p ≤ 0.0063). All scatter plots show data points from individual mice with overlaid group means ± s.e.m.; ^*^p < 0.05, ^**^p < 0.01, ^***^p < 0.001, based on Tukey’s post hoc test following ANOVA.

Using the third cohort of offspring (Supplementary Table 1), we compared ADHD-relevant dopaminergic and noradrenergic markers [51–55] among subgroups of MIA offspring relative to CON offspring in early adolescence (Fig. 3a). In this cohort, 47% (7 out of 15) of MIA offspring exhibited hyperactivity compared to CON offspring, whereas the remaining 53% (8 out of 15) did not (ANOVA: *F*_(2,19)_ = 20.13, *p* < 0.001; SUS-MIA vs. RES-MIA or CON: *p* < 0.001; Fig. 3b).

Subsequent comparisons of dopaminergic and noradrenergic markers in the mPFC of MIA subgroups and CON offspring revealed that only SUS-MIA offspring, but not RES-MIA offspring, displayed a significant decrease in TH immunoreactivity relative to CON offspring (*F*_(2,19)_ = 6.47, *p* < 0.01; SUS-MIA vs. CON: *p* < 0.01; SUS-MIA vs. RES-MIA: *p* < 0.05; Fig. 3c). SUS-MIA offspring also exhibited a significant increase in the DAT immunoreactivity in the mPFC compared to CON offspring (*F*_(2,19)_ = 7.67, *p* < 0.01; SUS-MIA vs. CON: *p* < 0.05; SUS-MIA vs. RES-MIA: *p* < 0.01; Fig. 3e). While no differences were observed in DBH immunoreactivity (Fig. 3d), RES-MIA offspring showed reduced NET immunoreactivity in the mPFC compared to both SUS-MIA and CON offspring (*F*_(2,19)_ = 7.80, *p* < 0.01; RES-MIA vs. CON: *p* < 0.05; RES-MIA vs. SUS-MIA: *p* < 0.01; Fig. 3f).

Analyses of the NAc revealed that TH immunoreactivity was significantly reduced in both SUS-MIA and RES-MIA offspring relative to CON offspring (*F*_(2,19)_ = 6.68, *p* < 0.01; RES-MIA vs. CON: *p* < 0.01; SUS-MIA vs. CON: *p* < 0.05; Fig. 3g). In contrast, DAT immunoreactivity in the NAc was selectively increased in SUS-MIA offspring compared to CON and RES-MIA offspring (*F*_(2,19)_ = 7.95, *p* < 0.01; SUS-MIA vs. CON: *p* < 0.05; SUS-MIA vs. RES-MIA: *p* < 0.01; Fig. 3h). No significant differences were observed in TH or DAT immunoreactivity in the CPu across subgroups (Fig. 3g,h).

Possible associations between dopaminergic and noradrenergic alterations and locomotor activity were examined using Pearson’s product-moment correlations, which were performed separately for CON and MIA offspring and corrected for multiple comparisons (significance threshold: *p* ≤ 0.0063). In CON offspring, locomotor activity positively correlated with DBH levels in the mPFC (*r* = 0.86, *p* < 0.005; Fig. 3i). This relationship was not observed in MIA offspring. Instead, locomotor activity in MIA offspring correlated negatively with TH levels in the mPFC (*r* = -0.74, *p* < 0.0015; Fig. 3i) and positively with DAT levels in the mPFC (*r* = 0.78, *p* < 0.0006; Fig. 3i) and NAc (*r* = 0.68, *p* < 0.005; Fig. 3i).

### Susceptible MIA offspring show distinct dopaminergic and noradrenergic alterations in cortical and subcortical brain regions during adulthood

**Fig. 4:**
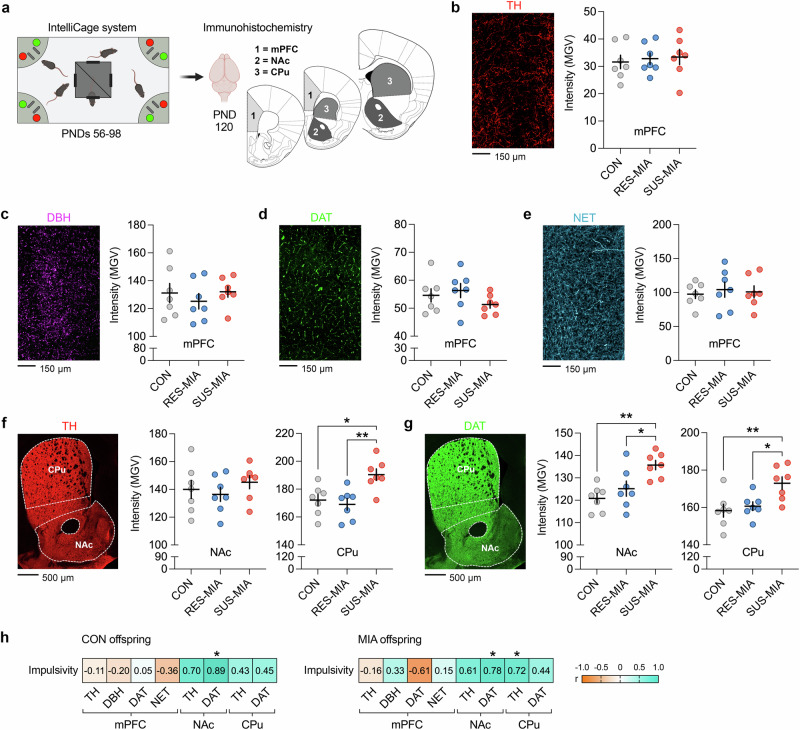
Adult dopaminergic and noradrenergic markers in control (CON) offspring and subgroups of offspring exposed to maternal immune activation (MIA). **a** Schematic representation of the experimental design. Dopaminergic and noradrenergic markers were analyzed in subgroups of adult MIA and CON offspring that had previously undergone testing in the IntelliCage system between postnatal days (PNDs) 56–98 (see Fig. 2). MIA offspring were stratified into resilient (RES-MIA) and susceptible (SUS-MIA) subgroups based on locomotor activity scores obtained prior to IntelliCage assessment (see Fig. 2). CON and MIA subgroups were subjected to immunohistochemical analyses of dopaminergic and noradrenergic markers in the medial prefrontal cortex (mPFC), nucleus accumbens (NAc), and caudate putamen (CPu) when they reached PND 120. **b** Representative immunofluorescence stain of tyrosine hydroxylase (TH) and TH intensity (mean gray value, MGV) in the mPFC. **c** Representative immunofluorescence stain of dopamine-β-hydroxylase (DBH) and DBH intensity (MGV) in the mPFC. **d** Representative immunofluorescence stain of dopamine transporter (DAT) and DAT intensity (MGV) in the mPFC. **e** Representative immunofluorescence stain of norepinephrine transporter (NET) and NET intensity (MGV) in the mPFC. **f** Representative immunofluorescence stain of TH and TH intensities (MGV) in the NAc and CPu. **g** Representative immunofluorescence stain of DAT and DAT intensities (MGV) in the NAc and CPu. **h** Pearson’s product-moment correlations between impulsivity (indexed by the total number of premature nosepoke responses in the operant delay-of-reinforcement task) and immunohistochemical markers for CON and MIA offspring. Negative and positive correlations are represented in orange and mint green color, respectively. Significant correlations are denoted with the symbol (*) after correction for multiple comparisons (significance threshold: ^*^p ≤ 0.0063). All scatter plots show data points from individual mice with overlaid group means ± s.e.m.; ^*^p < 0.05 and ^**^p < 0.01, based on Tukey’s post hoc test following ANOVA.

In view of our findings that susceptible MIA offspring exhibit distinct dopaminergic and noradrenergic alterations in cortical and subcortical brain regions during early adolescence, we next investigated whether these alterations persist into adulthood (Fig. 4a). To this end, we analyzed the same dopaminergic (TH and DAT) and noradrenergic (NET and DBH) markers in subgroups of adult MIA and CON offspring that had previously been tested in the IntelliCage system (second cohort of offspring; see Fig. 2).

In adult offspring, dopaminergic and noradrenergic markers in the mPFC did not differ between CON and MIA subgroups (Fig. 4b–e). However, adult SUS-MIA offspring displayed a significant increase in TH immunoreactivity in the CPu compared with adult RES-MIA and CON offspring (*F*_(2,18)_ = 7.81, *p* < 0.01; SUS-MIA vs. CON: *p* < 0.05; SUS-MIA vs. RES-MIA: *p* < 0.01; Fig. 4f). Moreover, SUS-MIA offspring showed a significant increase in DAT immunoreactivity in the NAc (*F*_(2,18)_ = 8.47, *p* < 0.01; SUS-MIA vs. CON: *p* < 0.01; SUS-MIA vs. RES-MIA: *p* < 0.05; Fig. 4g) and CPu (*F*_(2,18)_ = 6.47, *p* < 0.01; SUS-MIA vs. CON: *p* < 0.01; SUS-MIA vs. RES-MIA: *p* < 0.05; Fig. 4g) compared with RES-MIA and CON offspring.

Pearson’s correlations, performed separately for CON and MIA offspring and corrected for multiple comparisons (significance threshold: *p* ≤ 0.0063), revealed that impulsive behavior correlated positively with DAT immunoreactivity in the NAc in both CON *(r* = 0.89, *p* < 0.005, Fig. 4h) and MIA offspring (*r* = 0.78, *p* < 0.001; Fig. 4h). Moreover, impulsive behavior in MIA offspring, but not in CON offspring, correlated positively with TH immunoreactivity in the CPu (*r* = 0.72, *p* < 0.004; Fig. 4h). No other correlations reached statistical significance.

### Methylphenidate treatment normalizes locomotor hyperactivity and abnormal neuronal activation patterns in susceptible MIA offspring during early adolescence

**Fig. 5:**
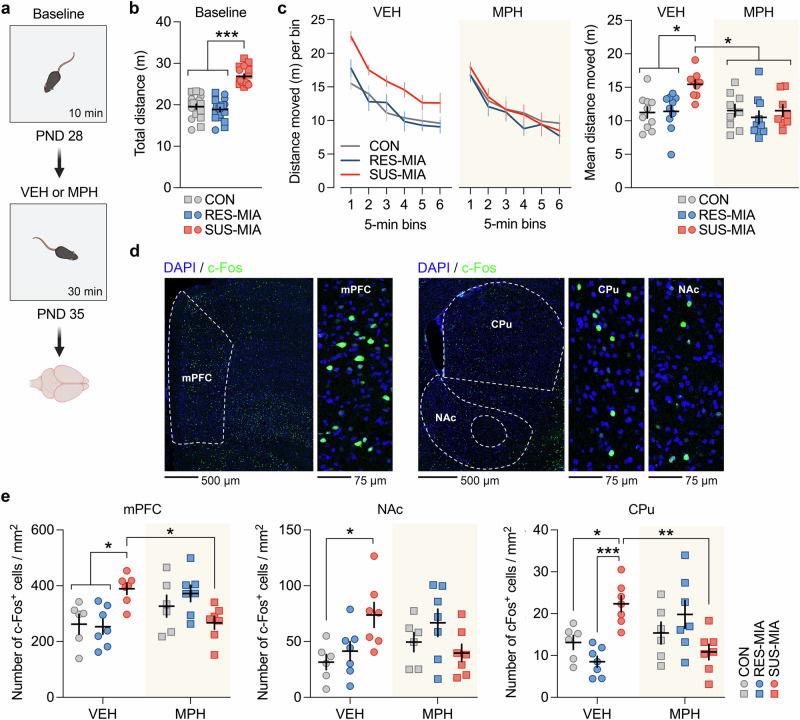
Effects of methylphenidate (MPH) treatment on locomotor activity and neuronal activation in control (CON) offspring and subgroups of offspring exposed to maternal immune activation (MIA). **a** Schematic representation of the behavioral and immunohistochemical testing sequence. On postnatal day (PND) 28, offspring were subjected to an open field test to stratify MIA offspring into resilient (RES-MIA) and susceptible (SUS-MIA) subgroups based on locomotor activity scores during early adolescence. The same CON and MIA subgroups were then assigned to vehicle (VEH) or MPH treatment and tested again in the open field on PND 35. Immediately following the open field test (corresponding to  90 min after MPH or VEH administration), brains were collected for immunohistochemical analyses of c-Fos protein to assess neuronal activation patterns. **b** Distance moved in the baseline open field test for CON and MIA subgroups, confirming the presence of locomotor hyperactivity in the SUS-MIA subgroup during early adolescence. Animals assigned to subsequent VEH and MPH treatment are denoted by circle and square symbols, respectively. **c** Line plots show the distance moved in 5-min bins during the open field test on PND 35 for CON and MIA subgroups receiving VEH or MPH, while scatter plots display the mean distance moved across the entire testing period. **d** Representative immunofluorescence images showing c-Fos (green) with DAPI counterstain (blue) in regions of interest, as indicated by dashed lines (mPFC: medial prefrontal cortex; NAc: nucleus accumbens; CPu: caudate putamen). **e** Quantification of c-Fos⁺ cells in the mPFC, NAc, and CPu of CON and MIA subgroups receiving VEH or MPH. All scatter plots show individual data points with overlaid group means ± s.e.m.; ^*^p < 0.05, ^**^p < 0.01, ^***^p < 0.001, based on Tukey’s post hoc test following ANOVA.

Using the fourth cohort of offspring (Supplementary Table 1), we next investigated whether MPH treatment is effective in normalizing locomotor hyperactivity in SUS-MIA offspring during early adolescence. The initial assessment of baseline locomotor activity in the open field on PND 28 (Fig. 5a) showed that 46% (17 out of 37) of MIA offspring exhibited hyperactivity compared to CON offspring, whereas the remaining 54% (20 out of 37) did not (ANOVA: *F*_(2,53)_ = 58.56, *p* < 0.001; SUS-MIA vs. RES-MIA or CON: *p* < 0.001; Fig. 5b). Based on these baseline measurements, CON, RES-MIA, and SUS-MIA offspring were then assigned to receive either VEH or MPH treatment to evaluate the effects of MPH on locomotor activity in a 30-min open field test (Fig. 5a). As shown in Fig. 5c, VEH-treated SUS-MIA offspring exhibited a consistent increase in distance moved throughout the entire testing period. This hyperactivity was normalized by MPH treatment, which did not alter locomotor activity in CON or RES-MIA offspring (Fig. 5c). RM-ANOVA of distance moved supported these findings, revealing significant main effects of bins (*F*_(5,250)_ = 75.06, *p* < 0.001), subgroup (*F*_(2,50)_ = 4.65, *p* < 0.05), and drug (*F*_(1,50)_ = 4.63, *p* < 0.05), as well as a significant two-way interaction between subgroup and drug (*F*_(1,50)_ = 3.87, *p* < 0.05). Post hoc analysis confirmed that VEH-treated SUS-MIA offspring differed significantly from all other conditions (all *p* < 0.05; Fig. 5c).

To examine patterns of neuronal activation in response to novelty exposure with or without additional MPH treatment, immunohistochemical evaluations of c-Fos expression in the mPFC, NAc, and CPu were conducted in a subset of animals that were subjected to the locomotor activity test in the open field (Fig. 5d). Paralleling the subgroup-specific patterns of locomotor activity, SUS-MIA offspring exhibited elevated c-Fos immunoreactivity across all three regions compared to VEH-treated CON and RES-MIA offspring (Fig. 5e), demonstrating heightened neuronal activation within these brain areas. MPH treatment normalized these increases, reducing c-Fos expression in SUS-MIA animals to levels comparable to VEH-treated CON offspring, while having no significant effect on CON or RES-MIA groups (Fig. 5e). These findings were supported by a significant two-way interaction between subgroup and drug (mPFC: *F*_(2,34)_ = 9.11, *p* < 0.001; NAc: *F*_(2,34)_ = 5.97, *p* < 0.01; CPu: *F*_(2,34)_ = 14.00, *p* < 0.001), as well as by post hoc analysis confirming significant differences between VEH-treated SUS-MIA offspring and the other conditions (see Fig. 5e).

## Discussion

Our study demonstrates that a subgroup of mice exposed to MIA displays key features of ADHD, including locomotor hyperactivity, impulsivity, and responsiveness to MPH treatment. These phenotypes parallel the hyperactive/impulsive ADHD type, which is characterized by excessive motor activity, impaired inhibitory control, and responsivity to MPH pharmacotherapy [15–18, 44, 46, 49, 57]. Longitudinal assessment of locomotor behavior revealed that subgroup-specific hyperactivity in MIA offspring was most pronounced during early adolescent stages but normalized to control levels by early adulthood. Although the mechanisms underlying this developmental trajectory remain to be elucidated, these findings align with clinical evidence indicating that motor hyperactivity in children with ADHD often declines across late adolescence [15, 18, 96, 97]. Despite normalization of the hyperactive phenotype, SUS-MIA offspring exhibited increased impulsive behavior when they reached early adulthood. These data align with the clinical representation of ADHD, showing that despite reductions in motor hyperactivity across adolescence, impulsive and attentional deficits often continue into adulthood [15, 18, 96, 97].

In addition to increased impulsivity, SUS-MIA offspring also exhibited reduced PPI in early adulthood, indicating persistent impairments in pre-attentive filtering. Our data replicate previous findings of subgroup-specific PPI deficits in mouse MIA models [9, 11, 12, 14] and indicate that PPI impairments are particularly pronounced in MIA-exposed offspring displaying locomotor hyperactivity during adolescence. However, this association does not imply that the overall PPI-disrupting effect of MIA, as previously reported in multiple independent studies [61, 92, 98–100], is absent or irrelevant. Indeed, when PPI was analyzed across all MIA offspring without subgroup stratification, a significant group-wide reduction in PPI relative to CON offspring was still observed, although statistical significance was reached only at pulse intensities of 110 and 120 dB_A_, and not at 100 dB_A_ (Supplementary Fig. S2). These findings align with our previous work demonstrating MIA-induced PPI deficits both at the whole-group level and following subgroup stratification [9]. Notably, the larger sample size used in our earlier study [9] likely enabled detection of significant overall PPI reductions across all pulse intensities.

While the presence of PPI impairments in individuals with ADHD remains a matter of debate [101–104], such deficits are well established in schizophrenia and related psychotic disorders [80, 105]. Based on the current findings, we interpret that the transient hyperactivity phenotype observed in a portion of MIA-exposed offspring may reflect subgroup-specific vulnerabilities that predispose to distinct but overlapping outcomes across the spectrum of neurodevelopmental and psychiatric disorders. Thus, alongside ADHD-like abnormalities, a subset of MIA offspring may also develop schizophrenia-related symptoms in late adolescence or adulthood. This interpretation aligns with epidemiological evidence showing that ADHD frequently co-occurs with other psychiatric and neurodevelopmental disorders, including schizophrenia, and that childhood ADHD is a risk factor for developing schizophrenia later in life [22–24]. In a broader framework, our findings are compatible with the emerging concept that: (1) one commonality across nosologically defined neurodevelopmental and psychiatric disorders is that underlying pathological processes begin during early development; (2) these disorders share environmental risk factors and molecular pathways; and (3) they may lie along a continuum of neurodevelopmental causalities, wherein the combination of genetic architecture and environmental context determines the specificity of eventual pathological outcomes [25–29].

By revealing subgroup- and brain region-specific alterations in catecholaminergic markers, our study highlights potential neuronal substrates underlying the variable behavioral outcomes in MIA offspring. Notably, these subgroup-specific neuronal alterations were age-dependent, consistent with the dynamic nature of MIA-induced neuropathologies [106, 107]. In SUS-MIA offspring, dopaminergic and noradrenergic alterations in the mPFC were evident during adolescence but largely normalized by adulthood. In contrast, several subcortical abnormalities including increased DAT and TH immunoreactivity in the NAc and/or CPu, were manifest specifically in adulthood. During adolescence, SUS-MIA offspring exhibited locomotor hyperactivity, which coincided with decreased TH levels in the mPFC and increased DAT levels in both the mPFC and NAc. Decreased TH in the mPFC may blunt the dopaminergic tone within prefrontal regulatory circuits, while increased DAT in the mPFC and NAc may accelerate dopamine clearance and reduce tonic dopaminergic signaling, thereby impairing inhibitory control and promoting hyperactivity [108–110]. The absence of these changes in RES-MIA offspring suggests that subgroup-specific dopaminergic dysregulation is a key determinant of variable locomotor outcomes following prenatal MIA exposure. In adulthood, impulsive behavior correlated positively with accumbal DAT levels in both CON and MIA offspring, consistent with prior studies linking high DAT expression in the NAc to impulsivity [109]. In adult SUS-MIA animals, impulsivity further correlated with increased TH in the CPu. These results are consistent with prior evidence linking elevated presynaptic dopaminergic activity in the CPu to impulsive behavior [111] and suggest that additional subcortical dopaminergic anomalies contribute to the emergence of impulsivity in SUS-MIA offspring. Collectively, our data indicate that subgroup- and brain region-specific alterations in catecholaminergic signaling may underlie variable behavioral phenotypes following prenatal MIA exposure, providing mechanistic insight into ADHD-related hyperactivity and impulsivity.

Building on these findings, some of the catecholaminergic alterations identified in SUS-MIA offspring resemble neuronal changes reported in individuals with ADHD. For example, reduced prefrontal TH expression in this subgroup parallels the decreased dopamine synthesis capacity in the PFC of ADHD subjects, as assessed using positron emission tomography with [fluorine-18]fluorodopa [112]. Moreover, elevated DAT expression in SUS-MIA offspring aligns with in vivo imaging findings of increased DAT availability in ADHD patients [51, 54, 83, 113]. It should be noted, however, that reports of increased DAT availability in ADHD are heterogeneous and conflicting, largely due to the impact of prior psychostimulant medication history [114–116]. Because our analyses of dopaminergic and noradrenergic markers were conducted in drug-naïve animals, potential confounding effects of previous drug exposure can be excluded in our study.

In addition to catecholaminergic alterations, we also found increased c-Fos expression in the mPFC, NAc, and CPu of SUS-MIA offspring displaying hyperactivity in the open field test, suggesting that these animals exhibit elevated neuronal activation in mesolimbic and corticostriatal circuits in response to novelty exposure. This pattern is consistent with findings from other rodent models relevant to ADHD [117, 118], including genetic mouse models [119], spontaneously hypertensive rats [120], Lister hooded rats [121], and rats with neonatal ventral hippocampal lesions [122]. The elevated c-Fos expression in SUS-MIA offspring may indicate increased sensitivity to novel stimuli that engage mesolimbic and corticostriatal circuits, a feature that parallels behavioral traits of the hyperactive/impulsive presentation of ADHD, such as heightened novelty-seeking and impulsivity [123]. Consistent with this, MPH treatment reduced both hyperlocomotion and c-Fos overactivation in SUS-MIA offspring, suggesting that normalization of cortico-striatal activity may underlie the therapeutic effects of MPH on novelty-induced hyperactivity in these animals.

While the primary focus of our study was the SUS-MIA group displaying hyperactivity, we would like to highlight the presence of distinct neuronal alterations emerging specifically in RES-MIA offspring. Extending our previous work demonstrating subgroup-specific transcriptomic alterations following prenatal MIA exposure [9, 11], the present study revealed a selective decrease in NET levels in the mPFC of RES-MIA offspring. These RES-MIA-specific changes may reflect counterregulatory or compensatory mechanisms that protect MIA offspring from developing overt behavioral alterations, such as hyperactivity or reward-related deficits. For example, because NET is responsible for clearing norepinephrine (NE) from the synaptic cleft, reduced NET expression is expected to result in higher extracellular NE levels in the mPFC [124]. In turn, elevated NE in this region can enhance inhibitory control over hyperactive circuits, stabilize cortical network activity, and modulate attention [124]. In RES-MIA offspring, this reduction in NET may therefore counterbalance potential excitatory or dopaminergic abnormalities, ultimately protecting them from developing hyperactivity. In contrast, SUS-MIA offspring lack this compensatory NET reduction and, in combination with reduced TH and increased DAT expression, may exhibit altered prefrontal dopamine signaling that manifests as hyperactivity. Based on these observations and our previous work [9, 11], future studies will be important to investigate whether RES-MIA-specific molecular and circuit-level changes can be leveraged in SUS-MIA offspring to ameliorate behavioral and cognitive abnormalities.

We acknowledge several limitations of our study. First, we did not assess another key feature of ADHD, namely attentional deficits [15–18]. While our focus was on hyperactivity, impulsivity, and related neuronal markers, deficits in sustained or selective attention are central to ADHD and remain unexplored here. However, previous studies in rodent and primate MIA models have reported attentional impairments [99, 125–129], but without stratifying offspring by hyperactivity status. Thus, it remains to be determined whether such deficits are generally induced by MIA or specific to the hyperactive subgroup. In addition, the present study did not include a detailed longitudinal investigation of PPI across early adolescence to adulthood. Therefore, developmental associations between MIA-induced hyperactivity phenotypes and pre-attentive processing remain to be examined in future studies. Repeated PPI testing may represent a considerable stressor for the animals, and in the present study, we aimed to focus primarily on the trajectory of locomotor hyperactivity while minimizing potential confounding factors induced by stress. Finally, we did not examine whether MPH could mitigate the deficits in PPI or impulsivity-related anomalies in SUS-MIA offspring displaying hyperactivity, emphasizing the need for future studies to determine whether these behavioral abnormalities respond to MPH pharmacotherapy.

Despite these limitations, we conclude that MIA in mice recapitulates features of ADHD in a subgroup of offspring, supporting the notion that MIA may be etiologically relevant only in a subset of individuals with ADHD. Notably, not all forms of MIA may increase the risk of ADHD in offspring, as suggested by a recent epidemiological study comparing distinct infectious and inflammatory exposures during pregnancy [130]. Given the substantial heritability of ADHD [131], future studies are needed to investigate how MIA interacts with genetic factors to shape phenotypes associated with ADHD. In view of the established links between ADHD, ASD and schizophrenia [19–26], it is also possible that the neurobehavioral alterations observed in SUS-MIA offspring reflect early neurobiological vulnerabilities that, in combination with additional genetic and environmental factors, may predispose to distinct but overlapping outcomes across the spectrum of neurodevelopmental and psychiatric disorders. Future longitudinal and mechanistic studies will be essential to determine whether the alterations identified in SUS-MIA offspring represent transient developmental perturbations specific to ADHD or precursors of broader psychopathological trajectories extending into other psychiatric disorders.

## Supplementary information

Supplementary Information

Supplementary MIA Reporting Guidelines

## Data Availability

All data needed to evaluate the conclusions in the paper are present in the main manuscript and Supplementary Information. All original data of this study are available from the corresponding author at reasonable request.
