# Supplementary material for: Maternal immune activation in mice recapitulates features of attention-deficit/hyperactivity disorder (ADHD) in susceptible offspring
Source: Neuropsychopharmacology. 2026 Feb 28;51(8):1497–510. doi: 10.1038/s41386-026-02373-7 (PMC13291228; doi:10.1038/s41386-026-02373-7)
Supplement: Supplementary file 1 — Supplementary Information [file 41386_2026_2373_MOESM1_ESM.pdf]

SUPPLEMENTARY INFORMATION

Maternal immune activation in mice recapitulates features of attention-deficit/hyperactivity disorder (ADHD) in susceptible offspring

Ron Schaer (MSc)[1], Nicole Wenger (MSs)[1], Sarah Steiner (DVM)[1], Tina Notter (PhD)[2,3], Urs Meyer (PhD)[1,3,#]

[1] Institute of Veterinary Pharmacology and Toxicology, University of Zurich, Zurich, Switzerland.
[2] Institute of Pharmacology and Toxicology, University of Zurich, Zurich, Switzerland.
[3] Neuroscience Center Zurich, University of Zurich and ETH Zurich, Zurich, Switzerland.

[#]Correspondence:
Prof. Urs Meyer (PhD)
Institute of Veterinary Pharmacology and Toxicology, University of Zurich,
Winterthurerstrasse 260, 8057 Zurich, Switzerland.
E-Mail: urs.meyer@vetpharm.uzh.ch
Tel.: +41 44 635 88 44

SUPPLEMENTARY MATERIALS AND METHODS

Animals

C57BL/6N male and female breeder mice (12 weeks old) were obtained from Charles River Laboratories (Sulzfeld, Germany) and housed in individually ventilated cages (IVCs; Allentown Inc., Bussy-Saint-Georges, France) as previously described [1]. Mice were maintained in a specific-pathogen-free (SPF) facility with controlled temperature (21 ± 3 °C) and humidity (50 ± 10 %) under a reversed 12 h light–dark cycle (lights off 9:00 AM–9:00 PM), with *ad libitum* access to standard chow (Kliba 3336, Kaiseraugst, Switzerland) and water. All procedures were approved by the Cantonal Veterinarian's Office of Zurich, Switzerland.

Breeding and maternal manipulations

Timed pregnancies were established via in-house breeding, initiated two weeks after animals acclimated to the facility. Male and female breeders underwent a timed-mating protocol as previously described [1–3]. Successful mating was confirmed by detection of a vaginal plug, after which females were single housed for the duration of pregnancy. The day a plug was observed was designated gestational day (GD) 0. Females exhibiting a plug on GD 0 and gaining ≥3 g by GD 12 were classified as pregnant [1–3].

On GD 12, pregnant mice were randomly assigned to a single intraperitoneal (i.p.) injection of low molecular weight (LMW) poly(I:C) (10 mg/kg; InvivoGen, Toulouse, France; cat.#: tlrl-picw, lot #PIW-41–05) or pyrogen-free 0.9 % NaCl vehicle (B. Braun, Melsungen, Switzerland), using an injection volume of 10 ml/kg. The quality, molecular composition, and immunopotency of this poly(I:C) lot were previously validated [3,4]. Immediately after injection, dams were returned to their home cages and left undisturbed until five days postpartum.

Four cohorts of dams were generated via identical on-site breeding procedures and maternal treatments (**Supplementary Table 1**). In cohort 1, $N = 8$ and $N = 12$ dams were assigned to vehicle (CON) and poly(I:C) (MIA) treatment, respectively. In cohort 2, $N = 8$ and $N = 18$ dams received CON and MIA treatment, whereas in cohort 3, $N = 6$ and $N = 10$ dams received CON and MIA treatment, respectively. Finally, cohort 4 comprised $N = 12$ and $N = 24$ dams treated with CON and

MIA, respectively. Overall, approximately twice as many MIA dams as CON dams were prepared, as we anticipated that about 50% of the offspring would be allocated to the RES-MIA and SUS-MIA subgroups, respectively, based on behavioral stratification [2]. Further methodological details on maternal manipulations are provided in the **Supplementary MIA Reporting Guidelines** [5].

Offspring of CON and MIA dams were weaned on postnatal day (PND) 21, during which they were ear-punched for permanent identification. Littermates of the same sex were segregated and housed in groups of 2-4 per cage, unless specified otherwise. From each CON or MIA litter (see above), one to two male offspring were randomly selected for inclusion in the experimental series. All offspring from each cohort first underwent an open field test to assess baseline locomotor activity (see below) and to stratify MIA offspring into susceptible (SUS-MIA) and resilient (RES-MIA) subgroups based on locomotor activity scores during early adolescence [2]. Offspring from cohort 1 (*n*(CON) = 12 and *n*(MIA) = 19) were used for longitudinal assessments of locomotor activity across early adolescence to adulthood, followed by a prepulse inhibition test of pre-attentive filtering in adulthood. Offspring from cohort 2 (*n*(CON) = 13 and *n*(MIA) = 26) were tested in the IntelliCage system to assess reward processing and impulsivity, followed by immunohistochemical analyses of dopaminergic and noradrenergic markers in adulthood. Offspring from cohort 3 (*n*(CON) = 7 and *n*(MIA) = 15) were used for immunohistochemical analyses of dopaminergic and noradrenergic markers in early adolescence. Offspring from cohort 4 (*n*(CON) = 19 and *n*(MIA) = 37) were used to compare the effects of methylphenidate (MPH) and vehicle (VEH) treatment on locomotor activity and c-Fos expression in early adolescence.

Supplementary Table 1. Overview of animal cohorts used in this study.

Cohort	Number of litters per group	Number of offspring per group	Tests
1	*N*(CON) = 8<br>*N*(MIA) = 12	*n*(CON) = 12<br>*n*(MIA) = 19	- Basal locomotor activity in the open field<br>- Longitudinal assessment of locomotor activity in the open field<br>- Prepulse inhibition test of pre-attentive filtering
2	*N*(CON) = 8<br>*N*(MIA) = 18	*n*(CON) = 13<br>*n*(MIA) = 26	- Basal locomotor activity in the open field<br>- Sucrose preference in the IntelliCage system<br>- Progressive ratio in the IntelliCage system<br>- Operant delay-of-reinforcement in the IntelliCage system

			- Immunohistochemical analyses of dopaminergic and noradrenergic markers
3	*N*(CON) = 6<br>*N*(MIA) = 10	*n*(CON) = 7<br>*n*(MIA) = 15	- Basal locomotor activity in the open field<br>- Immunohistochemical analyses of dopaminergic and noradrenergic markers
4	*N*(CON) = 12<br>*N*(MIA) = 24	*n*(CON) = 19<br>*n*(MIA) = 37	- Basal locomotor activity in the open field<br>- Locomotor activity in the open field after VEH or MPH treatment<br>- Immunohistochemical analyses of c-Fos expression after VEH or MPH treatment

Independent cohorts (cohorts 1–4) of timed-pregnant mice and their offspring were generated through on-site breeding. For each cohort, the table summarizes the number of litters (*N*) per treatment group (CON or MIA), the number of offspring (*n*) per group, and the behavioral or histological tests conducted in each cohort. From each CON or MIA litter, one to two male offspring were randomly selected for inclusion in the tests of interest. All offspring from each cohort first underwent an open field test to assess baseline locomotor activity and to stratify MIA offspring into susceptible (SUS-MIA) and resilient (RES-MIA) subgroups based on locomotor activity scores during early adolescence. Offspring from cohort 1 were used for longitudinal assessments of locomotor activity across early adolescence to adulthood, followed by a prepulse inhibition test of pre-attentive filtering. Offspring from cohort 2 were tested in the IntelliCage system to assess reward processing and impulsivity, followed by immunohistochemical analyses of dopaminergic and noradrenergic markers in adulthood. Offspring from cohort 3 were used for immunohistochemical analyses of dopaminergic and noradrenergic markers in early adolescence. Offspring from cohort 4 were used to compare the effects of methylphenidate (MPH) and vehicle (VEH) treatment on locomotor activity and c-Fos expression in early adolescence.

Locomotor activity tests

A standard open field test was used to assess baseline locomotor and exploratory activity in a novel environment [6]. The apparatus consisted of four identical open field arenas (dimensions: 40 × 40 × 35 cm; length × width × height) made from white polyvinyl chloride (OCB Systems Ltd., Hertfordshire, UK). These arenas were positioned in a testing room with evenly distributed, diffused lighting, providing approximately 30 lux at the center and 20 lux in the corners. An overhead digital camera monitored all four arenas simultaneously, recording at 5 Hz. The video data were analyzed using the EthoVision tracking system (Noldus Information Technology, The Netherlands). Each animal was gently placed in the center of its assigned arena and allowed to explore freely for either 10 min (to assess baseline locomotion) or 30 min (to evaluate the impact of MPH on locomotor

activity). For data analysis, the arena was conceptually divided into two zones: a central area (15 × 15 cm) and a surrounding peripheral zone. For each animal, the total distance moved in the entire arena, distance moved in the center zone, and time spent in the center zone were recorded and analyzed.

IntelliCage System

Reward processing and impulsivity were assessed using the IntelliCage system (TSE Systems, Bad Homburg, Germany), a fully automated home-cage apparatus designed to evaluate a wide range of behaviors in group-housed mice tagged with radio-frequency identification (RFID) microtransponders [7–10]. Because of microtransponder implantation (see below), the experiments using IntelliCage system started when the offspring reached early adulthood, i.e. PND 56.

The setup consisted of six polycarbonate cages (20.5 cm high; 58 × 40 cm top; 55 × 37.5 cm bottom; Techniplast, 2000P, Buguggiate, Italy), each equipped with four triangular operant test chambers (15 × 15 × 21 cm) integrated into the cage corners. Each operant chamber permitted the entry of a single mouse at a time and contained two drinking bottles accessible through round openings that could be opened and closed by motorized doors. The RFID antenna at each corner entrance recognized the microtransponder ID implanted in each mouse (see below), while infrared sensors and licking sensors inside the corner chambers monitored mouse behaviors. Visit duration was determined based on RFID signal detection combined with a temperature sensor confirming the animal’s presence within the corner. The rules governing door operation and the illumination of three LEDs positioned above each nosepoke hole were flexibly programmable to accommodate different behavioral paradigms using the TSE IntelliCagePlus Designer software.

Microtransponder implantation: On PND 56, mice were pretreated with carprofen (5 mg/kg, s.c.) and were anesthetized with isoflurane for subcutaneous microtransponder implantation (Virbac, Switzerland) between the scapulae. Correct placement and function of the transponder were confirmed using a microchip scanner. Post-surgical monitoring was performed for 48 hours, during which the injection site, body weight, and signs of pain or distress were closely observed.

Habituation to the IntelliCage system: Following recovery, mice were transferred from conventional IVCs to the IntelliCages in groups of 8-10 animals per cage and habituated for 7 days (PNDs 56-63). To reduce possible aggressive behavior, each cage contained three mouse houses and tissue paper, with additional tissue provided if fighting occurred, and mice were placed in the cages before 9:00 AM, during their resting period. During habituation, all corner doors in the operant chambers remained open, allowing free access to water from any corner, while standard chow (Kliba 3336, Kaiseraugst, Switzerland) was provided ad libitum via food hoppers positioned on top of the cages. Visits and licking behavior were continuously recorded using the IntelliCagePlus Controller software (TSE Systems). The last two days of habituation were used to determine baseline water intake for the subsequent sucrose preference test.

Nosepoke adaptation: After habituation, mice underwent a nosepoke adaptation phase for three consecutive days using a pre-programmed IntelliCagePlus Designer protocol. Upon entering a corner, each mouse was required to perform a single nosepoke at one of the two doors to open it for 10 s, allowing access to water. After 10 s, the door closed automatically, and the mouse needed to exit the corner to initiate a new trial. Each visit, nosepoke, and lick was continuously recorded to allow mice to learn the association between nosepoke responses and door opening prior to reward-based testing.

Sucrose preference test: Baseline water intake was recorded during the last two days of habituation (see above). One water bottle per corner was then replaced with 0.5% sucrose solution, with both water and sucrose freely available for 24 h. Bottle positions were counterbalanced across corners to control for side preferences, and visits and licking behavior were recorded using the IntelliCagePlus Controller. After 24 h, the sucrose and water bottles were swapped in each corner for an additional 24-h session, yielding 48 h of total testing. Sucrose preference (%) was calculated based on the total number of licks for water versus sucrose.

Progressive ratio test: Two corners were switched to 10% sucrose solution while the other two remained as water controls. The following progressive ratio reinforcement schedule was applied: 1, 2, 3, 4, 5, 6, 7, 8, 9, 10, 12, 14, 16, 18, and 20 nosepokes, with the mouse required to complete the specified number of nosepokes to open the sucrose door and obtain the reward. The

ratio increased to the next step once the mouse achieved ≥60% correct trials, as programmed in the IntelliCagePlus Designer. Each corner visit constituted a new trial, whereby successful completion of the nosepoke requirement opened the door for 7 s, while failure ended the trial and required the mouse to exit before starting a new one. This schedule continued for two weeks, allowing determination of each mouse's breakpoint, defined as the highest ratio of responses completed to obtain a reward before the animal ceased responding, as well as the total number of rewarded and non-rewarded corner visits. In addition, the number of rewarded responses for each animal was recorded and analyzed.

Operant delay-of-reinforcement test: Mice were first adapted to a green light stimulus provided by corner LEDs for three days. Two of the four corners contained 10% sucrose solution, while the remaining two corners contained water bottles. Water corners served as immediate-reward controls, with the green light activating upon entry and the door opening immediately after a nosepoke. When the animal entered a sucrose corner, a delay period was initiated before the green light was illuminated. The duration of this delay followed a predefined schedule, starting at 0.5 s during shaping and gradually increasing to 1, 2, 3, 4, 5, 6, 7, and 8 s. Any nosepoke performed during this reinforcement delay was recorded as a premature trial, indicating failure to wait for the cue, whereas a nosepoke after the light signaled success and opened the door for 7 s to allow drinking. This stepwise increase in delay allowed assessment of each animal's ability to tolerate progressively longer waiting periods before accessing the sucrose reward. Mice completing ≥ 60% of trials successfully were advanced to higher delay modules, enabling quantification of delay tolerance and operant response inhibition over a 14-day testing period.

Prepulse inhibition test for pre-attentative filtering

Pre-attentative filtering was assessed using a test of prepulse inhibition (PPI) of the acoustic startle reflex, which reflects a decrease in the magnitude of the startle reaction when a weak auditory stimulus (prepulse) precedes a sudden, more intense startle-inducing stimulus (pulse) by a short interval [11,12]. The assessment was carried out using four mouse startle chambers (San Diego Instruments, San Diego, CA, USA) as described before [1,13]. Each testing session involved a

sequence of discrete trials comprising four different trial types: pulse-alone trials, prepulse-plus-pulse trials, prepulse-alone trials, and no-stimulus trials (where only background noise was present). The pulse and prepulse stimuli consisted of abrupt increases in broadband white noise (lasting 40 ms and 20 ms, respectively), rising from a constant 65 dB_A background level, with a rise time of 0.2–1.0 ms. Stimulus intensities for the pulses were 100, 110, and 120 dB_A, and for the prepulses were 71, 77, and 83 dB_A. The interval between the onset of the prepulse and the onset of the pulse was fixed at 100 ms.

The PPI testing protocol followed previously validated procedures [3,13]. Mice were first placed in a Plexiglas holding cylinder within the chamber and given a 2-minute acclimation period before trials commenced. The session began with six consecutive pulse-alone trials, which were used to reduce variability in baseline startle responses and were excluded from the final analysis. Following this, animals were exposed to 10 blocks of test trials. Each block consisted of 16 trials: three pulse-alone trials (one per intensity), three prepulse-alone trials (+6, +12, or +18 dB_A), nine prepulse-plus-pulse combinations (3 prepulse × 3 pulse intensities), and one trial with no stimulus. Trials were delivered in a pseudorandom sequence with inter-trial intervals averaging 15 seconds (ranging between 10 and 20 seconds).

PPI for each pulse intensity (100, 110, or 120 dB_A) was calculated as the percent reduction in startle amplitude on prepulse-plus-pulse trials compared to pulse-alone trials using the following formula: 100 % × [1−(mean reactivity on prepulse-plus-pulse trials/mean reactivity on pulse-alone trials)]. This was computed for each prepulse intensity (+6, +12, or +18 dBA above background). In addition to PPI, the reactivity to pulse-alone trials was analyzed to examine possible group differences in acoustic startle reactivity *per se*.

Immunohistochemistry

Animals were deeply anesthetized with an overdose of pentobarbital (Esconarkon ad us. vet., Streuli Pharma AG, Switzerland) and transcardially perfused with 20 ml of oxygenated artificial cerebrospinal fluid (aCSF; pH 7.4), followed by 12 h post fixation in 4% phosphate-buffered paraformaldehyde (PFA) according to previous protocols [14,15]. The brains were cryoprotected in

30% sucrose in PBS for 48 h, frozen on dry-ice and stored at -80 °C. The brains were cut coronally with a sliding microtome at 30 µm (8 serial sections) and stored at − 20 °C in cryoprotectant solution (50 mM sodium phosphate buffer (pH 7.4) containing 15% glucose and 30% ethylene glycol; Sigma-Aldrich, Switzerland) until further processing.

Immunofluorescent stainings were performed according to previously established protocols [14,15]. In brief, brain sections were rinsed in Tris buffer (pH 7.4) before incubation with the primary antibodies. The following primary antibodies were used: rabbit anti-tyrosine hydroxylase (TH; Merck Millipore, cat. # AB152, 1:2,000), rabbit anti-dopamine-β-hydroxylase (DBH; Abcam, cat. # ab209487, 1:1,000), rat anti-dopamine transporter (DAT; Merck Millipore, cat. # MAB369, 1:1,000), mouse anti-norepinephrine transporter (NET; MAb Technologies Inc., cat. # NET05-2, 1:5,000), and rabbit anti-c-Fos (Santa Cruz Biotechnology, cat. # sc-52, 1:1,000). The primary antibodies were diluted in Tris buffer containing 0.2% Triton X-100 and 2% normal serum. The sections were incubated free-floating under constant agitation (100 rpm) overnight at 4 °C. On the following day, sections were washed 3 × 10 min in Tris buffer prior to a 30 min incubation period with an appropriate secondary antibody (Alexa488-conjugated anti-rabbit IgG, Molecular Probes, cat. # A-11008; diluted 1:1,000; Cy3-conjugated anti-mouse IgG, Jackson ImmunoResearch Laboratories, Europe Ltd, cat. # 715-165-150; diluted 1:1,000) and 4'6-diamidino-2 phenylindole dilactate (DAPI) (Thermo Fisher Scientific, Zurich, Switzerland, cat. # D3571; diluted 1:3000) in Tris-Triton containing 2 % normal serum at room temperature. After incubation, which was shielded from light, the sections were washed 3 × 10 min in Tris buffer, mounted onto gelatinized glass slides, coverslipped with Dako fluorescence mounting medium (S3023, Agilent, Switzerland), and stored in the dark at 4 °C until image acquisition (see below).

Microscopy and image analysis

An automated upright widefield slide scanning microscope (PhenoImager™ HT, Akoya Biosciences, Marlborough, USA) was used to acquire immunofluorescence-stained images using a 20× (air, NA 0.75) objective. For each animal, 4 to 5 sections containing the regions of interest were imaged.

TH, DAT, NET, and DBH immunoreactivity were quantified in brain areas receiving major dopaminergic and noradrenergic inputs [16–19], including mPFC (Bregma: +2.0 to +1.6 mm), nucleus accumbens (NAc; Bregma: +1.7 to +0.9 mm), and caudate putamen (CPu; Bregma: +1.2 to +0.4 mm). The density of c-Fos-immunoreactive cells were also quantified in these regions. However, given the sparse expression of DBH and NET in the CPu and NAc [20,21], these markers were only quantified in the mPFC, where they are abundantly expressed [22,23]. In each brain region of interest, the intensity of TH, DAT, NET, and DBH immunoreactivity was quantified by means of mean grey value (MGV) measurements using the ImageJ software, whereby a threshold was applied to remove background. The density of c-Fos-immunoreactive cells was assessed using QuPath (0.5.0-x64) for bioimage analysis. All image acquisition and quantification were conducted by an experimenter who was blinded to the treatment conditions.

SUPPLEMENTARY RESULTS

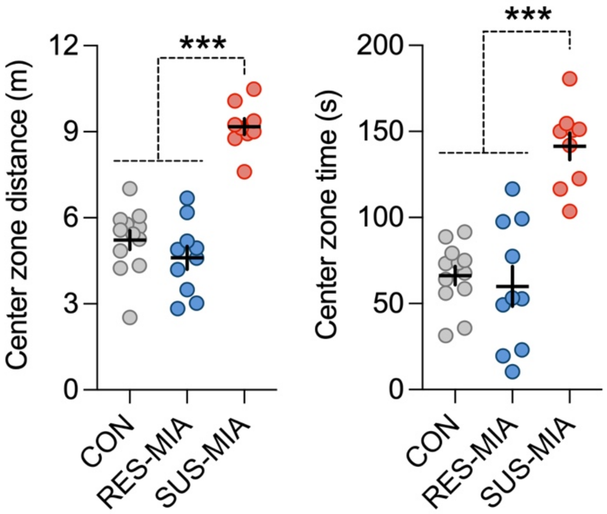

Supplementary Figure S1. Distance moved (m) and time spent (s) in the center zone of the baseline open field test conducted on postnatal day 28 in control (CON) offspring and offspring exposed to maternal immune activation (MIA) from cohort 1. MIA offspring were stratified into resilient (RES-MIA) and susceptible (SUS-MIA) subgroups based on differences in total distance moved (see *Fig. 1* in main text). All scatter plots show individual data points with overlaid group means ± s.e.m.; ***p < 0.001, based on Tukey's post hoc test following ANOVA (center zone distance: $F_{(2,28)} = 48.18$, $p < 0.001$; center zone time: $F_{(2,28)} = 26.92$, $p < 0.001$).

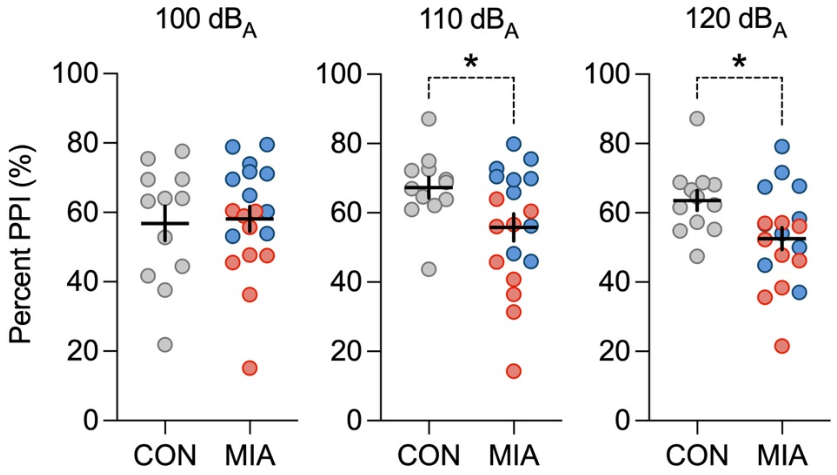

Supplementary Figure S2. Prepulse inhibition (PPI) in control (CON) offspring and non-stratified offspring exposed to maternal immune activation (MIA). The scatter plots show percent PPI as a function of different pulse intensities (100, 110, and 120 dBA) for individual CON and MIA offspring. Blue (resilient MIA offspring) and red (susceptible MIA offspring) colors in the MIA group denotes subgroup identity in stratified analyses (see *Figure 1* in main text). Note that when PPI was analyzed across all MIA offspring without subgroup stratification, a significant group-wide reduction in PPI relative to CON offspring was observed at pulse intensities of 110 ($t_{(29)}$ = 2.20, $p < 0.05$) and 120 dBA ($t_{(29)}$ = 2.38, $p < 0.05$), but not at 100 dBA. All data represent individual data points with overlaid group means ± s.e.m.

References

1. Mueller FS, Polesel M, Richetto J, Meyer U, Weber-Stadlbauer U. Mouse models of maternal immune activation: Mind your caging system! Brain Behav Immun. 2018;73:643–660.
2. Schaer R, Wenger N, Herrero F, Notter T, Meyer U. Ontogeny and plasticity of resilience and susceptibility in a mouse model of maternal immune activation. Brain Behav Immun. 2025;129:666–680.
3. Mueller FS, Richetto J, Hayes LN, Zambon A, Pollak DD, Sawa A, et al. Influence of poly(I:C) variability on thermoregulation, immune responses and pregnancy outcomes in mouse models of maternal immune activation. Brain Behav Immun. 2019;80:406–418.
4. Tillmann KE, Schaer R, Mueller FS, Mueller K, Voelkl B, Weber-Stadlbauer U, et al. Differential effects of purified low molecular weight Poly(I:C) in the maternal immune activation model depend on the laboratory environment. Transl Psychiatry. 2024;14:300.
5. Kentner AC, Bilbo SD, Brown AS, Hsiao EY, McAllister AK, Meyer U, et al. Maternal immune activation: reporting guidelines to improve the rigor, reproducibility, and transparency of the model. Neuropsychopharmacol Off Publ Am Coll Neuropsychopharmacol. 2019;44:245–258.

6. Belzung C, Griebel G. Measuring normal and pathological anxiety-like behaviour in mice: a review. Behav Brain Res. 2001;125:141–149.
7. Kiryk A, Janusz A, Zglinicki B, Turkes E, Knapska E, Konopka W, et al. IntelliCage as a tool for measuring mouse behavior - 20 years perspective. Behav Brain Res. 2020;388:112620.
8. Iman IN, Yusof NAM, Talib UN, Ahmad NAZ, Norazit A, Kumar J, et al. The IntelliCage System: A Review of Its Utility as a Novel Behavioral Platform for a Rodent Model of Substance Use Disorder. Front Behav Neurosci. 2021;15:683780.
9. Nigri M, Bramati G, Steiner AC, Wolfer DP. Appetitively motivated tasks in the IntelliCage reveal a higher motivational cost of spatial learning in male than female mice. Front Behav Neurosci. 2024;18:1270159.
10. Lipp H-P, Krackow S, Turkes E, Benner S, Endo T, Russig H. IntelliCage: the development and perspectives of a mouse- and user-friendly automated behavioral test system. Front Behav Neurosci. 2024;17:1270538.
11. Braff DL, Geyer MA, Swerdlow NR. Human studies of prepulse inhibition of startle: normal subjects, patient groups, and pharmacological studies. Psychopharmacology (Berl). 2001;156:234–258.
12. Swerdlow NR, Weber M, Qu Y, Light GA, Braff DL. Realistic expectations of prepulse inhibition in translational models for schizophrenia research. Psychopharmacology (Berl). 2008;199:331–388.
13. Schaer R, Mueller FS, Notter T, Weber-Stadlbauer U, Meyer U. Intrauterine position effects in a mouse model of maternal immune activation. Brain Behav Immun. 2024;120:391–402.
14. Notter T, Coughlin JM, Gschwind T, Weber-Stadlbauer U, Wang Y, Kassiou M, et al. Translational evaluation of translocator protein as a marker of neuroinflammation in schizophrenia. Mol Psychiatry. 2018;23:323–334.
15. Notter T, Schalbetter SM, Clifton NE, Mattei D, Richetto J, Thomas K, et al. Neuronal activity increases translocator protein (TSPO) levels. Mol Psychiatry. 2021;26:2025–2037.
16. Roessner V, Sagvolden T, Dasbanerjee T, Middleton FA, Faraone SV, Walaas SI, et al. Methylphenidate normalizes elevated dopamine transporter densities in an animal model of the attention-deficit/hyperactivity disorder combined type, but not to the same extent in one of the attention-deficit/hyperactivity disorder inattentive type. Neuroscience. 2010;167:1183–1191.
17. Miller EM, Pomerleau F, Huettl P, Russell VA, Gerhardt GA, Glaser PEA. The spontaneously hypertensive and Wistar Kyoto rat models of ADHD exhibit sub-regional differences in dopamine release and uptake in the striatum and nucleus accumbens. Neuropharmacology. 2012;63:1327–1334.
18. Somkuwar SS, Kantak KM, Dwoskin LP. Effect of methylphenidate treatment during adolescence on norepinephrine transporter function in orbitofrontal cortex in a rat model of attention deficit hyperactivity disorder. J Neurosci Methods. 2015;252:55–63.

19. Kantak KM. Rodent models of attention-deficit hyperactivity disorder: An updated framework for model validation and therapeutic drug discovery. Pharmacol Biochem Behav. 2022;216:173378.
20. Cimarusti DL, Saito K, Vaughn JE, Barber R, Roberts E, Thomas PE. Immunocytochemical localization of dopamine-beta-hydroxylase in rat locus coeruleus and hypothalamus. Brain Res. 1979;162:55–67.
21. Schroeter S, Apparsundaram S, Wiley RG, Miner LH, Sesack SR, Blakely RD. Immunolocalization of the cocaine- and antidepressant-sensitive l-norepinephrine transporter. J Comp Neurol. 2000;420:211–232.
22. Miner LH, Schroeter S, Blakely RD, Sesack SR. Ultrastructural localization of the norepinephrine transporter in superficial and deep layers of the rat prelimbic prefrontal cortex and its spatial relationship to probable dopamine terminals. J Comp Neurol. 2003;466:478–494.
23. Ranjbar-Slamloo Y, Fazlali Z. Dopamine and Noradrenaline in the Brain; Overlapping or Dissociate Functions? Front Mol Neurosci. 2019;12:334.
